# Design of a Novel Road Pavement Using Steel and Plastics to Enhance Performance, Durability and Construction Efficiency

**DOI:** 10.3390/ma14030482

**Published:** 2021-01-20

**Authors:** Wei Jiang, Dongdong Yuan, Aimin Sha, Yue Huang, Jinhuan Shan, Pengfei Li

**Affiliations:** 1 Key Laboratory for Special Area Highway Engineering of Ministry of Education, Chang’an University, Xi’an 710064, China; ddy@chd.edu.cn (D.Y.); ams@chd.edu.cn (A.S.); jhshan@chd.edu.cn (J.S.); 2020021080@chd.edu.cn (P.L.); 2 School of Highway, Chang’an University, Xi’an 710064, China; 3 Institute for Transport Studies (ITS), University of Leeds, 34–40 University Road, Leeds LS2 9JT, UK; Y.Huang1@leeds.ac.uk

**Keywords:** asphalt pavement, durability, mechanical properties, asphalt, steel, plastic

## Abstract

Durability is one important problem that pavement engineers need to address in pavement’s long service life. Furthermore, easily recycled pavement materials, and safe and efficient pavement construction are also important areas for development in road engineering. For these reasons, a new asphalt steel plastic (ASP) pavement structure was proposed with an asphalt mixture forming the surface layer, and steel plate and plastic materials functioning as the main load-bearing layers. Based on a comprehensive performance review and cost-benefit analysis, stone mastic asphalt (SMA) is recommended to be used as the surface layer; and A656 steel plate and acrylonitrile butadiene styrene (ABS) plastic materials should be the main load-bearing layer, on top of a foundation layer made with graded crushed stones. A glass fiber reinforced polymer (GFRP) insulation layer is recommended for use between the steel plate and ABS. Mechanical properties of the ASP pavement were analyzed using the finite element method. Laboratory tests were conducted to verify the thermal insulation performance of GFRP, the high-temperature stability and the fatigue resistance of ASP pavement. Results show that some of the mechanical properties of ASP pavement (with a structure of 80 mm SMA asphalt mixture, 8 mm steel plate, 140 mm ABS and 200 mm crushed stones) are comparable with conventional long-life pavement (with 350 mm asphalt layer overlaying 400 mm graded crushed stones). Dynamic stability of the ASP slab specimens can reach 10,000 times/mm, and the fatigue life is about twice that of SMA. Besides, the ASP pavement can be prefabricated and assembled on-site, and thus can greatly improve construction efficiency. From the lifecycle perspective, ASP pavement has many advantages over traditional pavements, such as durability, lower environmental footprint and recyclability, making it is worth further research.

## 1. Introduction

Safety, durability and construction efficiency of road infrastructure are the key objectives pursued by civil engineers and researchers [1,2,3].

Road infrastructures, especially the pavement materials are exposed to the natural environment [4,5], under effects of ultraviolet light [6], oxygen [7], water and temperature cycles [8,9], leading to binder aging, permanent deformation and cracking. In the meantime, the pavement’s surface is subject to repeated vehicle loads, leading to material failure in the forms of rutting and fatigue cracking [10]. Besides, the surface of the pavement is worn after a certain period of use [11,12], resulting in reductions in texture depth and skid resistance [13,14]. Therefore, the pavement’s surface is considered the primary functional layer, which needs to be periodically reconstructed by milling, recycling or overlaying every 5 to 10 years [15,16]. The concept of long-life pavement or perpetual pavement refers to the main structures of the pavement [17,18], such as the base and subbase, which are expected to maintain good performance and not have structural damage during the service period [19,20]. Different countries and regions have different standards for long life pavement, but usually require a service life of more than 40 years without major structural strengthening [21,22,23].

Road infrastructure, vehicles and traffic management all influence road safety. Durable pavement structure helps to reduce the extent and frequency of maintenance work, which is often associated with disruption to road users [24,25]. In the meantime, reduced maintenance needs and improved construction efficiency, such as through prefabrication, can reduce health and safety risks to road workers exposed to the traffic [26,27], and reduce congestion and fuel consumption [28,29]. In this sense, the durability achieved by using a novel pavement structure contributes to improving safety, improving construction efficiency and a reduction in the environmental footprint [30,31]. Since the beginning of asphalt pavement construction, civil engineers and researchers have been working on the durability, safety and construction efficiency of road infrastructure [32]. Most of these works are associated with the structural design and performance improvement of road materials.

To enhance durability, the asphalt mixture is improved by optimizing aggregate skeleton grading [33], and adopting SBS (styrene-butadiene-styrene) modified asphalt with good high and low-temperature performance [34,35,36]. The fatigue resistance of pavement is improved by increasing the thickness of the asphalt layer, and adopting a high modulus asphalt mixture base [37,38,39,40]. Additionally, warm mixing and cold mixing are used to reduce the carbon and harmful gas emissions in the construction [41,42,43,44]. Construction efficiency is improved through automation and mechanized operations, including mix production, paving and compaction [45,46]. Furthermore, prefabrication is welcome for its quality assurance, ability to speed up construction and ability to reduce the exposure of workers.

In addition to research on traditional pavement materials and structural design, some cutting-edge experimental work has been carried out in recent years. For example, self-healing capsules are added to asphalt pavement materials, which are filled with polymer healing agents [47]. Cracks in the pavement structure will break the capsules and release the healing agents to repair the cracks [48]. In other studies, steel velvet, steel fiber or steel slag have been added to the asphalt mixture, which is heated regularly by external induction heating [49]. During heating, the micro-cracks of the pavement materials can be filled to extend the service life. Research and applications of prefabricated concrete pavement and rollpave asphalt pavement are also ongoing, to improve construction efficiency and quality [50,51]. In recent years, research on intelligent transportation has been carried out worldwide; the key objectives are to improve road durability, transport efficiency and safety [52,53].

The main challenges to achieving the above objectives can be summed up as follows: (1) Material degradation. The deterioration of pavement due to the combined effects of asphalt aging, repeated load, moisture egress, freeze–thaw cycle, etc. [54,55]. (2) Construction quality. The quality of asphalt pavement construction is affected by many factors, such as the temperature of mixing, paving and compaction, particle segregation, etc. [56,57]. In general, it is difficult to ensure a consistently high quality of pavement construction using current quality control in the manufacture, transport and site operations. (3) Vehicle overload. Overloading leads to early damage to the road and jeopardizes the safety of road users, and this problem is particularly severe in developing countries. Traditional pavement materials and structures are fundamentally vulnerable to premature failure caused by overloading [58,59,60,61]. With the ongoing developments in materials science and construction technology, it is imperative to think of whether alternative materials should be used to pave the road, which will substitute asphalt and concrete materials, providing better performance, improved durability, enhanced safety and superior construction efficiency. Nouali et al. [62] examined the suitability of using plastic bag waste in asphalt mixture. The results indicated that adding plastic waste can increase asphalt mixture’s stiffness modulus and water resistance substantially. Bhardwaj et al. [63] summarized the plastic materials used in flexible pavements. The results showed that the use of waste polyethylene for road construction as a replacement of a limited percentage of asphalt can increase the strength of the flexible pavement, and it will also be beneficial for the environment. Murugan [64] presents the research conducted on the samples of traditional asphalt mixes and asphalt mixes with plastic granules as a replacement for the coarse aggregate. The results indicated that the use of e-plastic particles as aggregate replacements in the bitumen mix seems feasible. Jiao et al. [65] studied the utilization of steel slag in asphalt concrete. The results showed that the steel slag can improve the thermal conductivity of asphalt concrete. Cabalar [66] investigated the utilization of zinc coated steel CNC (computer numerical control) milling waste for road pavement subgrade. The results indicated that CNC spirals can be considered as an alternative soil reinforcement technique for construction of road pavement subgrade. The above research has made a lot of effort in looking for alternative pavement materials, but the use of plastic, steel and other materials is very limited.

Therefore, this paper explores the possibility of using plastic plate and steel plate in the pavement structural layers. We describe the components of the ASP (asphalt steel plastic) pavement structure and its design principles; we developed a finite element analysis (FEA) model to calculate the strain and deflection within ASP pavement; we verified the ASP pavement’s performance (rutting resistance performance) and durability (fatigue resistance performance) based on an indoor test, and evaluated the feasibility of applying this new pavement structure on a large scale from the perspectives of performance and cost. The research proposes a new concept of pavement design, which is fundamentally different from the traditional approach. The key criteria, such as durability, construction efficiency and environmental footprint for this new pavement structure were also evaluated. The rest of this paper is organized as follows. Section 2 starts with describing the ASP pavement’s design principles, structure layers and materials for structure layers. Section 3 presents the mechanical properties of ASP pavement by FEA. Section 4 studies the rutting resistance performance and fatigue resistance performance of ASP pavement. Section 5 analyzes ASP pavement’s cost evaluation and recyclability. The conclusion and further study are summarized in Section 6.

## 2. Design of a New Road Pavement

### 2.1. Design Principle and Structure Layers

**Figure 1:**
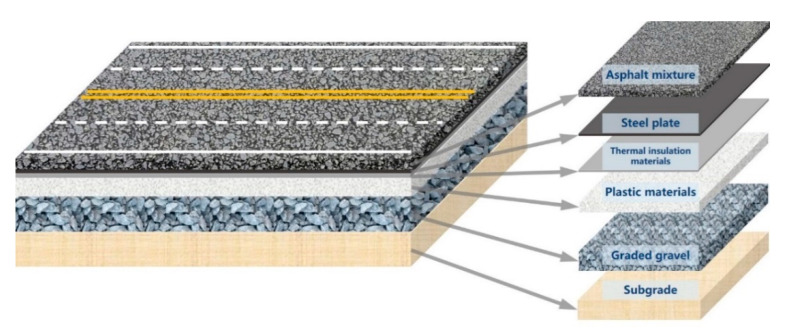
Asphalt steel plastic (ASP) pavement structure.

Aligned with the core principle of pavement durability and serviceability, the new pavement consists of two parts: (1) the surface functional layer, which has 5–10 years’ service life with good skid resistance and riding quality; (2) the load-bearing structure layer, which provides a stable and durable structure for taking vehicle load. Furthermore, materials of both layers should come with adequate temperature stability, water stability, aging resistance, and being renewable or reusable at the end of design life. Based on the above requirements, the pavement materials and structure are designed from bottom to top as illustrated in Figure 1.

(1) First layer: graded gravel. Made of loose particles without binder, the graded gravel layer is located on top of a compacted subgrade that has good deformation resistance. On the one hand, it can effectively spread the stress transmitted from upper structure layers; on the other hand, as a regulating layer, it can shield off the impact of water and temperature change of the subgrade on upper pavement structure. Besides, the graded gravel layer can provide a stable and level working platform for constructing upper layers.
(2) Second layer: plastic materials. The lower base of pavement is made of plastic polymer with great mechanical strength, chemical stability, resistance to shock, wear and heat, easy to process at a reasonable cost. In the new pavement structure, the plastic layer is used as the base for bearing. In comparison to the traditional pavement, the plastic layer can be prefabricated in a factory and using recycled plastic as raw materials. Moreover, the use of prefabricated plastic layer can improve construction efficiency and effectively reduce environmental pollution.
(3) Third layer: thermal insulation. It uses functional materials with good thermal insulation and resistance to high temperature. During pavement construction, the temperature can reach about 150–180 °C and in the service period, the temperature of the pavement surface is often 60 °C or even higher [67]. Considering that plastic materials are easy to deform and age at high temperature, it is important to have a thermal insulation layer on the plastic materials, to reduce the heat transfer downward from the pavement surface.
(4) Fourth layer: steel plate. Steel is nearly isotropic, having advantages of high strength, plasticity and toughness, extremely durable, easy to form and process, and able to take a static and dynamic load of vehicles. The steel plate and the plastic material layer are the main load-bearing structure layers. Considering the high price of steel, a thin steel plate should be adopted as far as sufficient stability and durability can be met. In the new pavement structure, the steel plate layer serves the intelligent pavement as the functional layer, and it can be used as the load-bearing layer to disperse the pavement force because of its high stiffness. Compared to the traditional pavement, the steel plate can be manufactured through prefabrication in the factory. Additionally, the steel plate can be used as the carrier of intelligent components to serve the future smart road.
(5) Fifth layer: the surface functional layer. Considering that asphalt pavement provides good driving comfort, and the high efficiency of construction, milling and recycling compared to concrete pavement, this study adopted asphalt mixture as the surface functional layer.

In conclusion, the asphalt steel plastic (ASP) pavement is mainly composed of asphalt mixture, steel plate and plastic materials as shown in Figure 1. To ensure that the structural layers of the pavement are continuous which can bear and transfer loads as a whole, a good bond needs to be present between layers.

### 2.2. Materials for the Structure Layer

Based on the above-mentioned concept of pavement design, the following structural layers and component materials are selected.

Graded gravel is used for the sub-base (foundation) and asphalt mixture for the surface functional layer; these are the same as in traditional pavement. The gradation of the graded gravel is shown in Table 1.

The plastic materials used in the base course consist of organic polymer materials with resin as the main component, and plasticizer, filler, lubricant and colorant as additives, which can flow and form into shape under certain temperature and pressure in the process. Due to the difference in raw materials and processing technology, the physical and mechanical properties of the plastics are also quite different. It is, therefore, necessary to select the suitable materials for the pavement structure layer that have the required stress and strain characteristics.

When selecting plastic materials for a load-bearing structural layer of the pavement, it is necessary to consider many factors, such as the mechanical properties (modulus, bending strength and fatigue resistance), chemical stability (aging resistance, corrosion resistance and flame retardant), the temperature in the construction process and of the pavement in the service period, in addition to cost, environmental impact and recyclability. Based on these requirements, acrylonitrile butadiene styrene (ABS), polyoxymethylene (POM) and polyethylene terephthalate (PET) are all worth considering.

The thermal insulation layer can prevent or reduce the heat transfer from the pavement surface to the plastic base during the construction, thus avoiding the softening and deformation of the plastic base. Glass fiber reinforced polymer (GFRP) sheet that has the advantages of slow heat transfer, good thermal insulation and corrosion resistance, can be considered for this purpose.

Steel plate and plastic materials are the main load-bearing layers of the new pavement. From the perspective of cost and practicability, low alloy steel plates should be used, such as A656 (ASTM, USA) [68], S355J2 (EN, European norm) [69], Q345D (China, GB) [70]. To improve the bond between the steel plate and the upper and lower layer, the surface can be roughened by grinding, shot blasting or sand-blasting during the forming of the steel plate. At the same time, considering that water may corrode the steel plate during the long service life of the pavement, anti-corrosion and anti-rust paint (e.g., zinc-rich epoxy paint) can be applied on both sides of the steel plate to ensure the long-term performance of the steel.

The asphalt mixture of surface functional layer is required to have good surface performance. Stone Mastic Asphalt (SMA), Hot Rolled Asphalt (HRA), Porous Asphalt (PA) and other asphalt mixtures can be considered. The same criteria for selecting the mixture type apply, such as skid resistance, riding comfort and cost, with additional consideration for bond strength with the steel plate underneath.

A bond should be provided between each structural layer to ensure that all layers perform integrally. Epoxy resin and epoxy asphalt with good adhesive force can be used. Generally, the thermal insulation performance of epoxy resin is better than that of epoxy asphalt, thus epoxy resin was used as the binding material between the GFRP and upper (steel plate) and lower (ABS) layer, which is helpful to reduce the downward heat transfer during construction and service life. Epoxy asphalt can be used to bind steel plate and SMA materials, as the requirement for thermal insulation is less stringent.

**Figure 2:**
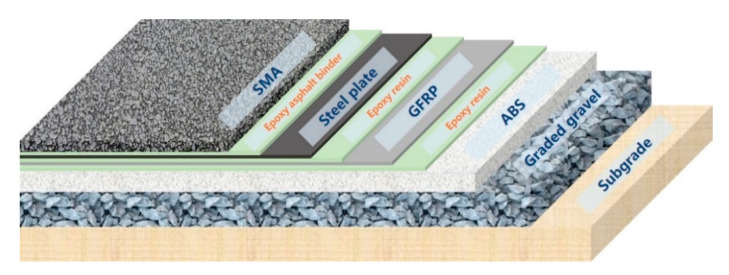
Proposed materials for ASP pavement structural layer.

Based on the above, the pavement structure as shown in Figure 2 is selected for mechanical test and simulation analysis. Among them, the SMA mixture consists of SBS modified asphalt (the technical specification of asphalt is shown in Table 2), diabase as coarse aggregate (apparent relative density is 2.943 g/cm^3^), and limestone as fine aggregate (apparent relative density is 3.086 g/cm^3^). The mixture gradation and asphalt content are shown in Table 3.

## 3. Simulation of Mechanical Properties of ASP Pavement

This section analyzed the mechanical properties of ASP pavement with different thicknesseses, evaluated the influence of layer thickness on the pavement performance, and compared them with conventional asphalt pavement.

### 3.1. Thickness and Materials of the Pavement Structure

The selected thickness of ASP pavement surface layer is 4, 6, 8 cm, steel plate layer is 0.6, 0.8, 1.0, 1.2 cm, ABS layer is 12, 14, 16, 18, 20 cm, graded crushed stone layer is 20 cm, soil subgrade is 200 cm. The thickness of the GFRP layer is relatively small, about 0.5 cm. To simplify the model, the GFRP layer is not considered in the stress-strain calculation. Table 4 shows the thicknesses in the ASP pavement model, which includes a total of 60 pavement structures with different thicknesses combinations of SMA (×3), steel (×4), and ABS (×5). In subsequent analysis, it is expressed in the form of a-b-c (a represents the thickness of ABS, cm; b represents the thickness of steel plate, cm; c represents the thickness of asphalt mixture layer, cm). For example, 16-0.6-6 means the thicknesses of asphalt mixture surface, steel plate and ABS layer are 6 cm, 0.6 cm and 16 cm, respectively. The thickness of a typical asphalt pavement structure is shown in Table 5 for comparison.

### 3.2. Mechanical Model and Parameters

**Figure 3:**
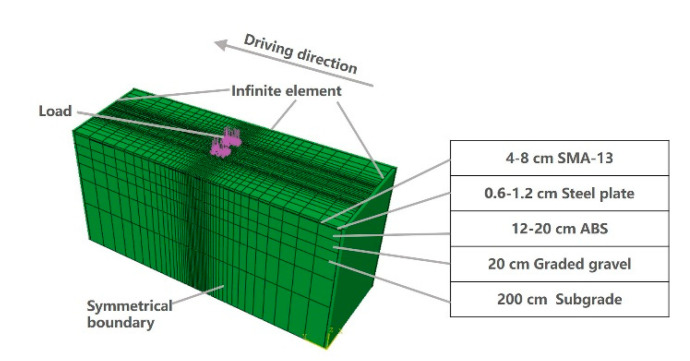
ASP pavement finite element model.

The Standard Module of finite element analysis software Abaqus (version 6.14) (Pawatucket, RI, USA) is used to analyze the mechanical properties of ASP pavement. The second-order solid structure element (3D, 20-node) of hexahedron is simulated in the model, with a physical dimension of 1.75 m × 5 m, as seen in Figure 3. The surface grid is transited from a 2.5 cm × 2.5 cm dense grid to a 0.3 m × 0.6 m sparse grid. The boundary condition is along the traffic direction, which is assumed to be infinite, and infinite elements are used at both ends. The road width used infinite element at one end and symmetry constraint at the other end. The bottom of pavement is assumed to be a fixed end, and there is no displacement or rotation in x, y and z directions (x represents the traffic, y represents the road width and z represents the road depth) [71]. The pavement structure model is shown in Figure 3. Load on the pavement surface is applied to a rectangular-shaped area, which is modeled to be a 0.24 m × 0.15 m area of a dual circular uniform load. Under the standard axle load of 100 kN, the ground stress under the tire is a uniform value of about 0.7 MPa. The mechanical parameters of pavement materials are shown in Table 6.

### 3.3. Stress–Strain Response of Each Material

**Figure 4:**
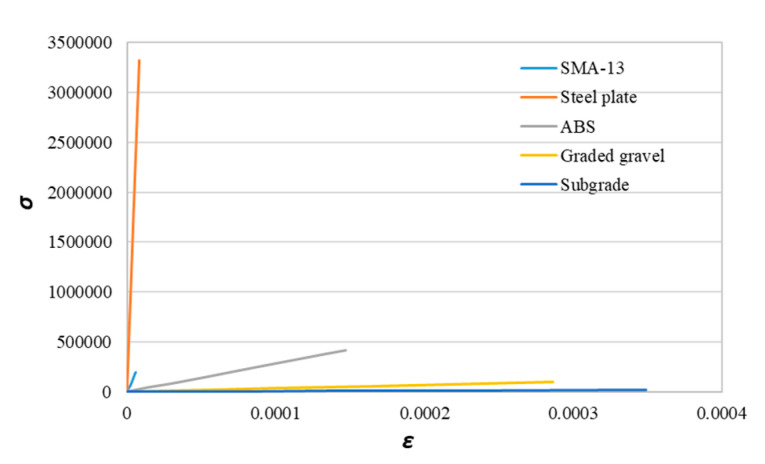
Stress–strain response of each material.

SMA, steel plate, ABS, graded gravel and subgrade are modeled respectively. The finite element analysis model is described in Section 3.2. The mechanical parameters of SMA, steel plate, ABS, graded gravel and subgrade are given in Table 6. The thickness of SMA, steel plate, ABS, graded gravel and subgrade are 4 cm, 0.6 cm, 12 cm, 20 cm, 200 cm, respectively. Figure 4 presents the stress-strain response of each material (SMA, steel plate, ABS, grade gravel and subgrade). It is can be seen that each material belongs to elastic deformation under the standard axle load of 100 kN. When the stress is removed, the deformation disappears. The stress is proportional to the strain of each material, which satisfies the Hooke’s law. The slope of the stress-strain curve represents the elastic modulus.

### 3.4. Mechanical Properties of the Pavement Structure

**Figure 5:**
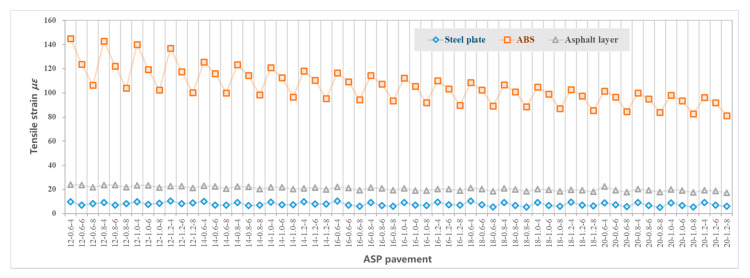
Tensile (horizontal) strain at the bottom of each layer of ASP pavement with different thicknesses.

**Figure 6:**
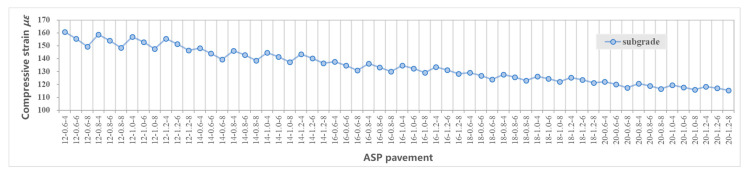
Compressive (vertical) strain at the top of subgrade (note: compressive (vertical) strain is negative).

**Figure 7:**
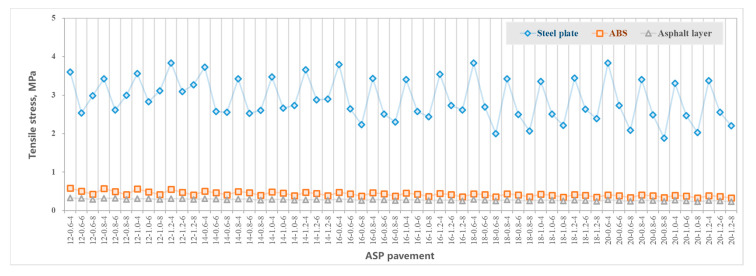
Tensile stress at the bottom of each layer of ASP pavement with different thicknesses.

**Figure 8:**
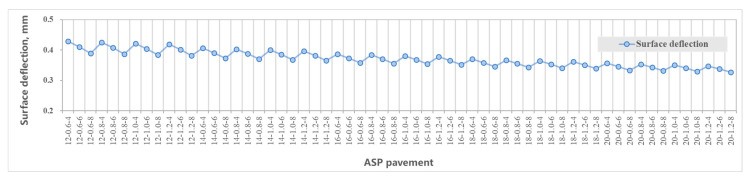
Surface deflection of ASP pavement.

Figure 5 shows the maximum tensile strain at the bottom of layers including the asphalt layer, steel plate layer and ABS layer. Figure 6 shows the maximum compressive strain at the top of the subgrade. Figure 7 shows the maximum tensile stress at the bottom of the asphalt layer, steel plate layer and ABS layer. Figure 8 shows the surface deflection of the pavement. In Figure 5, Figure 6, Figure 7 and Figure 8, results are presented for different thicknesses of the pavement.

#### 3.4.1. Asphalt Mixture Surface Layer

Asphalt mixture surface layer is directly subject to vehicle load. According to Figure 5, the thickness of the asphalt layer increases from 4 cm to 8 cm, which has little effect on the tensile stress at the bottom of the asphalt layer. The impact of the steel plate and ABS thickness on the tensile stress at the bottom of the asphalt mixture layer is also limited. The calculated range of tensile stress is 0.235–0.327 MPa.

Similarly, the range of tensile strain at the bottom of asphalt layer as a result of the difference in layer thickness is limited, according to Figure 5. The calculated range of tensile strain of the 60 structures is 1.738 × 10^−5^~2.420 × 10^−5^.

Surface deflection is the vertical deformation of pavement surface caused by vehicle load, which reflects the overall stiffness of pavement structure. The surface deflection value decreases with an increase in structural layer thickness, according to Figure 8. The effect of the thickness of the asphalt layer and ABS layer on reducing the deflection value is more significant than the thickness of the steel plate layer. The calculated range of vertical deflection in the middle of the wheel (of the dual circular vertical uniform load) of the 60 structures is 33~43 (0.01 mm).

#### 3.4.2. Steel Plate Layer

Steel plate is the material with the highest modulus, and the layer with the maximum tensile stress in the ASP pavement structure. According to Figure 6, the tensile stress at the bottom of the steel plate layer is mainly related to the thickness of the asphalt mixture layer and steel plate layer. The changes in ABS thickness have no significant effect on the tensile stress. Generally, when the ABS layer is thicker than 16 cm, with the increase of asphalt mixture layer thickness from 4 cm to 8 cm, the tensile stress at the bottom of the steel plate layer decreases. When the asphalt layer is between 4 cm and 6 cm, with the increase of steel plate thickness, the tensile stress at the bottom of the steel plate layer first decreases and then increases. Generally, when the steel plate thickness is between 0.8 cm and 1 cm, the tensile stress at the bottom of the steel plate layer reaches the minimum value. When the asphalt layer is 8 cm thick and the ABS is 12–18 cm, with the increase of steel plate thickness, the tensile stress at the bottom of the steel plate layer increases. The calculated range of tensile stress of the 60 structures is 1.885~3.834 MPa, far less than the allowable tensile stress of steel plate of 174 MPa.

The tensile strain at the bottom of the steel plate is mainly affected by the thickness of the asphalt mixture layer, which generally decreases with an increase of the asphalt layer thickness, according to Figure 5. However, due to the good bending ability of the steel plate, the tensile strain at the bottom of the steel plate is relatively small. The calculated tensile strain range of the 60 structures is 5.150 × 10^−6^~1.047 × 10^−5^, which is far less than the allowable strain of steel plate of 8.44 × 10^−4^.

#### 3.4.3. ABS Layer

ABS layer is underneath the steel plate layer, and the bottom tensile stress is affected by the thickness of the steel plate, asphalt mixture layer and ABS layer. Generally, the bottom tensile stress of the ABS layer decreases with an increase of asphalt mixture layer thickness, steel plate thickness and ABS layer thickness, according to Figure 7. The calculated bottom tensile stress range of the 60 structures is 0.329~0.588 MPa, which is far less than the allowable tensile stress of ABS of 24.5 MPa.

According to Figure 5, the maximum tensile strain of the ASP structure appears at the bottom of the ABS layer. The tensile strain of the ABS layer decreases with an increase of asphalt mixture layer thickness, steel plate thickness and ABS layer thickness. The range of tensile strain at the bottom of ABS of the 60 structures is 8.121 × 10^−5^~1.450 × 10^−4^, which is far less than the allowable strain of ABS of 1.11 × 10^−2^.

#### 3.4.4. Subgrade

**Figure 9:**
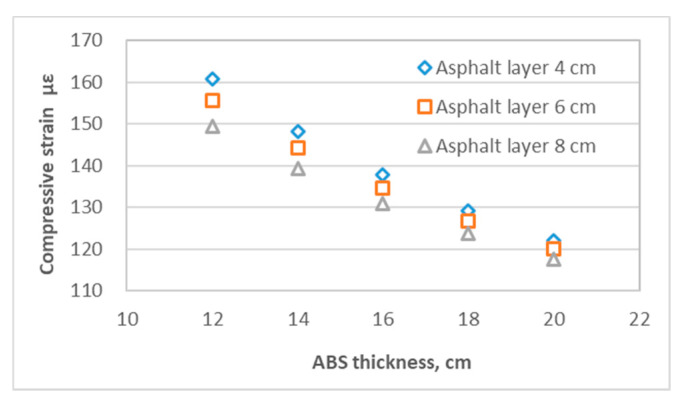
Influence of asphalt mixture layer and ABS layer thickness on the vertical compressive strain at subgrade (the thickness of the steel plate layer is fixed at 0.6 cm).

Vertical compressive strain at top of the subgrade is closely related to the thickness of the structural layers, so that it decreases with an increase of the overall thickness of the asphalt mixture layer, steel plate and ABS, according to Figure 6. The thickness of ABS layer and asphalt mixture layer have a significant effect on the compressive strain at subgrade, with an increase of the thickness of ABS layer from 12 cm to 20 cm, the effect of a thicker asphalt layer on reducing the compressive strain at subgrade decreases, as shown in Figure 9. The calculated range of vertical compressive strain at subgrade of the 60 structures is 1.153 × 10^−4^~1. 608 × 10^−4^.

#### 3.4.5. Comparison between ASP and Traditional Pavement

Table 7 compares the mechanical performance of the 14-0.8-8 ASP with the traditional pavement. Due to the difference in structure layer thickness and materials, the calculated mechanical properties are not always comparable. However, the stress and strain of the asphalt mixture layer in the ASP is lower than those in traditional pavement structure: the tensile strain at bottom of the layer is 73% lower, and the tensile stress at bottom of the layer is 13% lower. ASP pavement uses steel plate and ABS materials as the main load-bearing layers which have good mechanical properties. The calculated stress and strain are far less than the allowable for these materials, which indicates that the structural layer has sufficient bearing capacity and fatigue resistance.

Compared with the traditional pavement, the vertical compressive strain at top of the subgrade of ASP pavement increases by 65% and the surface deflection increases by 31%. The reason is that compared with traditional pavement structure (75 cm), the ASP pavement is thinner (between 36.6 cm and 49.2 cm), and the vertical distance for load transfer and distribution is shorter. The vertical compressive strain at top of the subgrade can be reduced by increasing the thickness of the graded crushed stone layer.

In conclusion, ASP pavement (with a structure of 8 cm asphalt mixture layer, 0.8 cm steel plate layer, 14 cm ABS and 20 cm graded crushed gravel) can provide desirable mechanical properties and take the required vehicle load. The design of ASP pavement is similar to the traditional pavement, except the requirements for pavement materials and thickness corresponding to traffic levels are different. When the pavement is designed for lower traffic levels, the thickness of the ASP pavement can be further reduced.

## 4. Indoor Test and Evaluation of ASP Pavement

### 4.1. Thermal Insulation of GFRP

**Figure 10:**
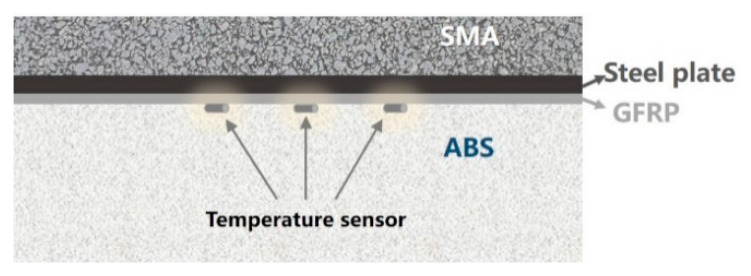
Schematic of the temperature sensor layout in the test piece.

**Figure 11:**
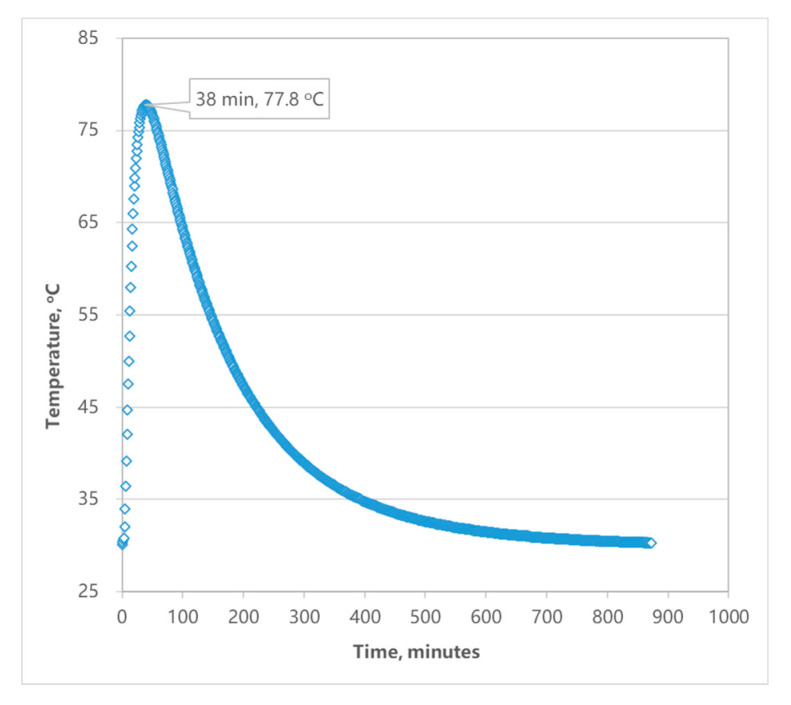
Average temperature of ABS’s surface.

The temperature of paving and rolling asphalt mixture can reach about 170 °C, and the melting point of ABS material is about 120 °C. To verify the effectiveness of GFRP thermal insulation layer and to measure the temperature of ABS during construction, the thermal insulation performance of GFRP was tested in the laboratory with slab specimens (dimension: 300 mm length × 300 mm width × 100 mm thickness, structure: 70 mm SMA, 5 mm steel plate, 5 mm GFRP and 20 mm ABS). Three temperature sensors are embedded on top of the ABS layer (the distance between the temperature sensors is 50 mm), and the temperature during the indoor test was recorded every minute, as shown in Figure 10. The average temperature of the ABS surface collected by sensors is shown in Figure 11.

The initial temperature of the SMA mixture was 170 °C, the temperature recorded by sensors in the ABS rose rapidly when the specimen is formed, and reached the peak temperature of 77.8 °C at 38 min. After that, the temperature started to decrease. Figure 11 show that the temperature of the ABS surface under the thermal insulation layer is halved compared with that on the thermal insulation layer. It indicated that the GFRP thermal insulation layer can effectively reduce heat transfer to the ABS layer, and thus ensure that the ABS plastic is at a reasonable temperature during pavement construction.

During the long service life of the pavement, the maximum temperature of the asphalt pavement surface is generally about 60~70 °C in summer. It can be therefore assured that, the temperature of the ABS layer will be far less than the allowable temperature of the material through the insulation by steel plate and GFRP.

### 4.2. Rutting Resistance of ASP

**Figure 12:**
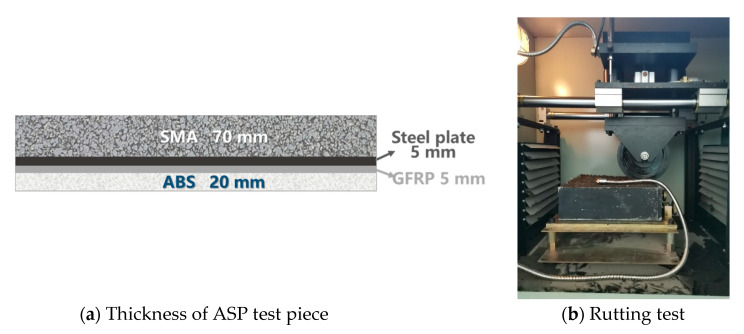
Rutting test of ASP.

The ASP test piece is 300 mm × 300 mm × 100 mm by size (Figure 12a), and the arrangement of the rutting test is shown in Figure 12b, according to JTG E20-2011 (China) [75]. To simulate the resistance of pavement structure to rutting, the test pieces compose of multi-layer materials with the structure of 70 mm SMA, 5 mm steel plate, 5 mm GFRP and 20 mm ABS. The epoxy asphalt binder layer is set between SMA and steel plate, and the epoxy resin layer is set between GFRP and steel plate in the upper layer and ABS plate in the lower layer. The total thickness of the above structure including adhesive layers is 100 mm. Due to the scale effect, the thickness of the layers is different from that recommended in Section 3.4.5, and the thicknesses of the steel plate and ABS layer are reduced accordingly. To accurately evaluate the performance of ASP pavement, the thickness of the asphalt mixture surface layer is 70 mm (it is close to the recommended thickness). Considering that the steel plate and thermal insulation are rigid layers without plastic deformation, the thicknesses of steel plate layer and thermal insulation layer were reduced as half-scale. The thickness of ABS layer is reduced as one seventh scale. The use of reduced scale will make the experimental results relatively small.

The dynamic stability of ASP slab specimens is shown in Table 8. Due to the use of SMA asphalt mixture which has good high-temperature stability, and steel plate and ABS which can take the high load, the dynamic stability of ASP specimens can reach 10,000 times/mm. In reference [76], the results showed that the dynamic stability of ordinary SMA-13 is 3365 times/mm. The dynamic stability of ASP pavement is much greater than that of ordinary SMA-13, which indicates that the ASP structure has good high-temperature stability.

### 4.3. Fatigue Resistance of ASP

**Figure 13:**
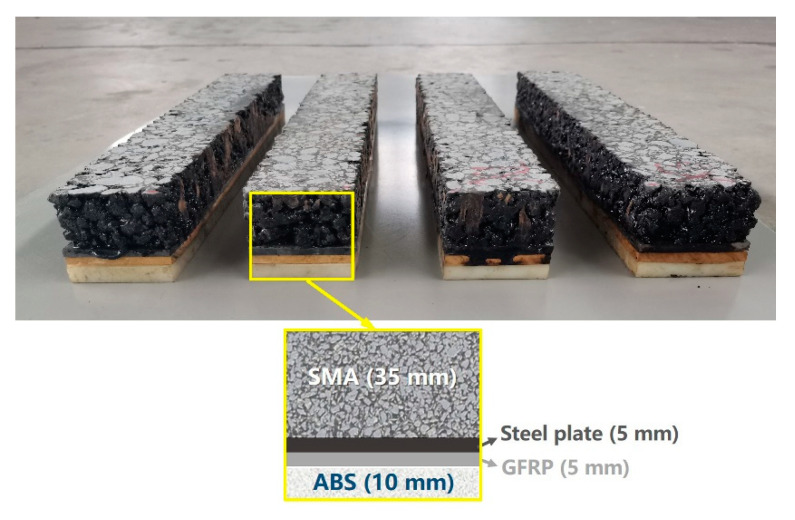
The specimen of ASP beam for fatigue testing.

As shown in Figure 13, the size of the beam specimen for fatigue test is 400 mm (length) × 63.5 mm (width) × 55 mm (height), according to AASHTO T321-17 (USA) [77] and JTG E20-2011 [75]. The structure of the ASP specimen is composed of 35 mm SMA, 5 mm steel plate, 5 mm GFRP and 10 mm ABS. The epoxy asphalt binder layer is set between SMA and steel plate, and the epoxy resin layer is set between GFRP and steel plate in the upper layer and ABS plate in the lower layer. Comparison is made with SMA of the same size. The test was carried out on pneumatic independent four points bending fatigue testing machine, and the temperature was 20 °C, the loading frequency was 10 Hz and the control strain was 1000 μm. The maximum and minimum fatigue loads were 5 kN and 0 kN, respectively. The flexural stiffness modulus corresponding to 50 times of repeated loading was taken as the initial flexure stiffness modulus, and the loading time when the flexural stiffness modulus was reduced to 50% of the initial flexure stiffness modulus is defined as the fatigue life of the specimen. The flexure stiffness modulus is calculated as

(1) $$S=\frac{\sigma_{t}}{\varepsilon_{t}}$$

where *S* is the flexure stiffness modulus, Pa; $\sigma_{t}$ is the maximum tensile stress, Pa; $\varepsilon_{t}$ is the maximum tensile strain, m/m.

(2) $$\sigma_{t}=\frac{L\times P}{w\times h^{2}}$$

where $\sigma_{t}$ is the maximum tensile stress, Pa; *L* is the beam span, m; *P* is the peak load, N; *w* is the breadth of beam, m; *h* is the height of beam, m.

(3) $$\varepsilon_{t}=\frac{12\times \delta \times h}{3\times L^{2}-4\times a^{2}}$$

where $\varepsilon_{t}$ is the maximum tensile strain, m/m; δ is the maximum strain of beam center, m; *a* = 0.119 m.

**Figure 14:**
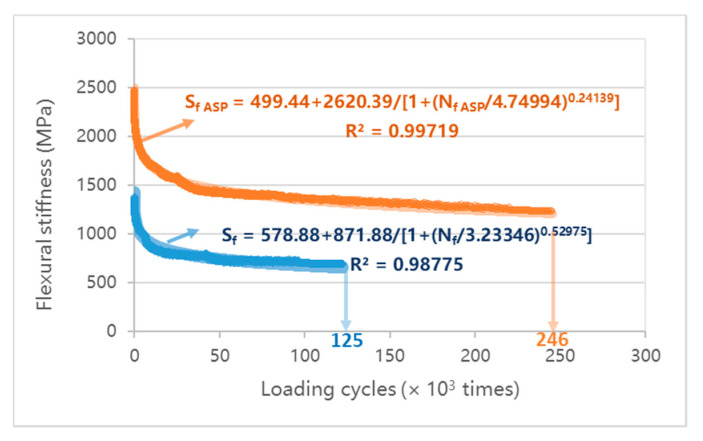
Curve of flexural stiffness with the loading times.

**Figure 15:**
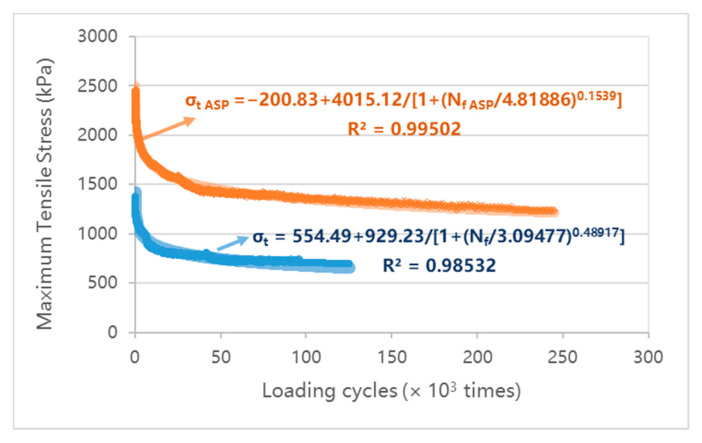
Curve of maximum tensile stress with the loading times.

Figure 14 presents the curve of the flexural stiffness modulus with the loading times. According to the above definition of fatigue failure, the initial flexural stiffness modulus of the ASP specimen after 50 times of repeat loading is 2458 MPa and it takes 2.46 × 10^5^ times of loading to decrease the flexural stiffness modulus to 50 % of the initial flexure stiffness modulus. The initial flexural stiffness modulus and fatigue life of the SMA specimen are 1378 MPa and 1.25 × 10^5^ times, respectively. Under the same strain level, the fatigue life of an ordinary asphalt mixture is less than 4 × 10^4^ times [78]. Figure 15 displays the curve of the maximum tensile stress in the middle of the beam with the loading times, it can be seen that the maximum tensile stress of ASP specimen varies from 2.4 MPa to 1.2 MPa at the same strain level (1000 μm), which is significantly higher than that of SMA specimen (1.4 MPa to 0.7 MPa). The results show that, under the same (controlled strain) loading conditions, although the maximum tensile stress of the ASP specimen is about 2 times that of the SMA specimen, the ASP specimen still shows a good fatigue life, which is about 2 times that of the SMA specimen. This is because of the homogeneity and integrity of ABS and steel plate materials, and the high elastic modulus of steel plate, ASP pavement have a high elastic ratio and better fatigue resistance.

**Figure 16:**
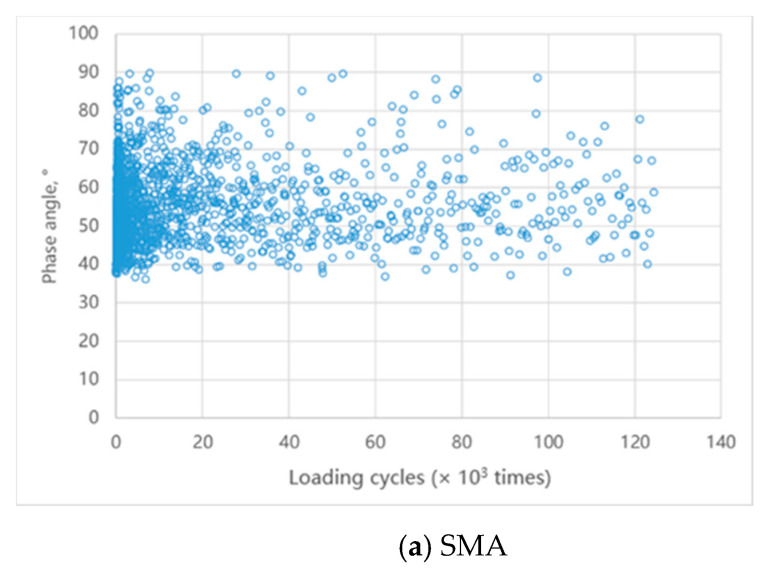

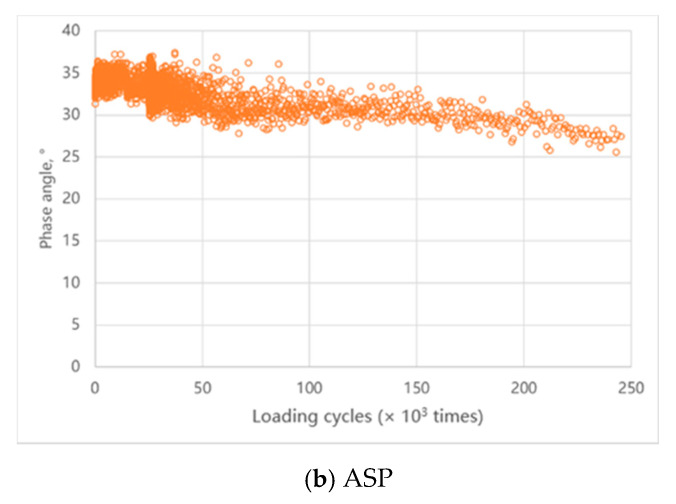
Phase angles of specimens during loading.

Figure 16 illustrates the change of the phase angle of the specimen during loading. The phase angle is the lag effect of strain on stress of the material under loading, which reflects the proportion of viscoelastic components. The phase angle is 0° when the material is completely elastic, and 90° when the material is fully viscous. It can be seen that the phase angle of the SMA specimen varies greatly (Figure 16a), ranging from 35° to 90°. The phase angle of the ASP specimen is largely between 25° to 40° (Figure 16b), and the overall trend shows a slight decrease as the loading cycles increase. The phase angle results also prove that the ASP pavement has a higher elastic ratio and better fatigue resistance.

**Figure 17:**
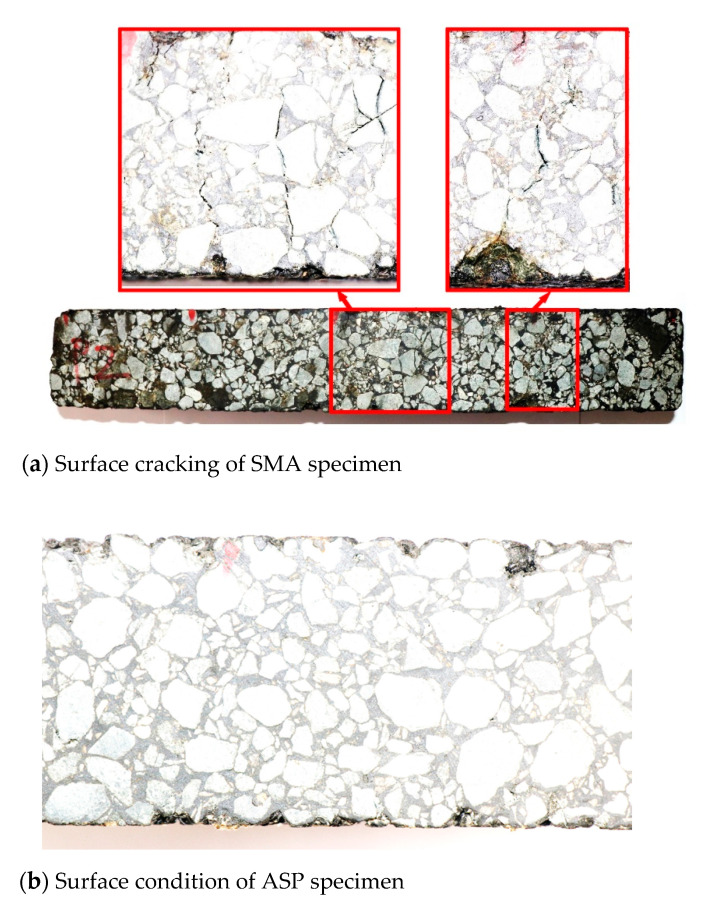

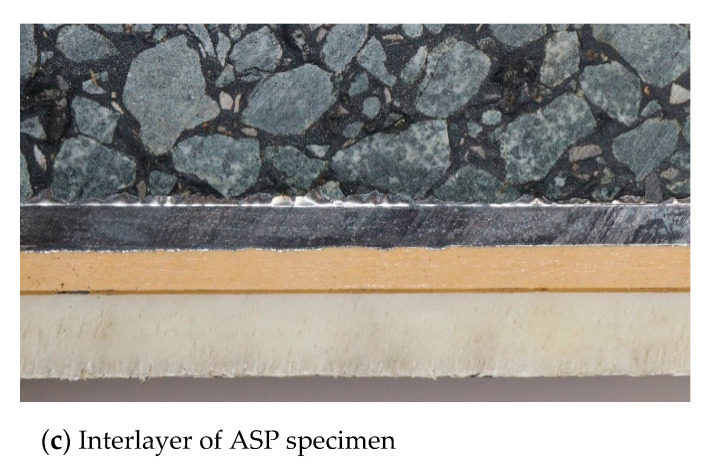
Surface condition of SMA and ASP specimens after loading.

By observing the surface condition of the specimens, the crack development on the surface of the SMA specimen is obvious after loading, as shown in Figure 17a. By comparison, there is no obvious crack on the surface of the ASP specimen, as shown in Figure 17b. Furthermore, ABS, GFRP, and steel plate are closely bonded without cracking at interlayer, as shown in Figure 17c. This observation also proves that the ASP pavement with epoxy asphalt and epoxy resin as the interlayer bonding materials exhibited great adhesion strength after about 250,000 times of 1000 μm strain loading.

## 5. Cost Evaluation and Recyclability

**Figure 18:**
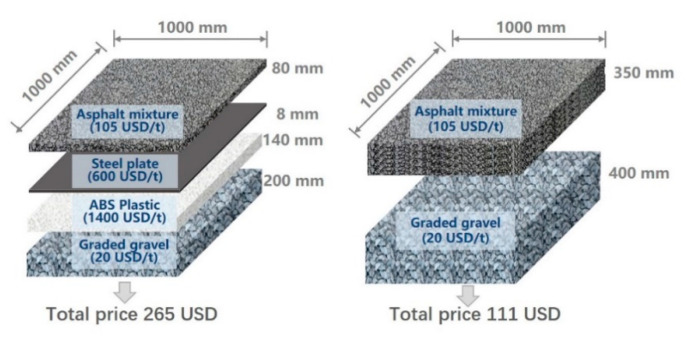
Comparison of cost for ASP and a traditional pavement structure.

Based on a set pavement structure (80 mm asphalt mixture, 8 mm steel plate, 140 mm ABS and 200 mm graded gravel), the cost of 1 m^2^ ASP pavement materials is calculated to be about 265 USD. For comparison, the cost of 1 m^2^ traditional pavement materials (with 350 mm asphalt overlaying 400 mm graded crushed stones) is 111 USD. Due to the difference in material prices between regions, the cost of ASP is estimated to be about 2~3 times of traditional pavement. It can be calculated from Figure 18 and Table 6 that, ABS accounts for a large proportion (86%) of the material cost. However, the cost may be reduced when the plastic plate is manufactured in large quantities and using recycled plastic as raw material. Due to the low density of ABS and the thin ASP pavement structure, the pavement self-weight per unit area is greatly reduced, to only about 50% of traditional pavement reducing the transportation cost and foundation requirement. Besides, through prefabrication in factory, the construction period can be greatly shortened, reducing the cost and the carbon emissions associated with construction activities on site.

Generally speaking, ASP pavement will increase the material cost, but the cost of transportation and construction onsite will be reduced, the construction efficiency and product quality will be improved as well. On the other hand, the plastic plate and steel plate used in the pavement can be recycled at the end of the service life. For instance, the steel plate coated with anti-corrosion and anti-rust paint can be recovered nearly at 100% at the end of service life. On the supply chain, both plastic plates and steel plates can use recycled materials in manufacturing. Therefore, considering the durability, construction efficiency, recycling and environmental footprint, the additional cost may be acceptable from the lifecycle perspective.

Table 9 compares the ASP pavement with traditional pavement indicating its advantages in efficiency, durability and environmental footprint.

Additionally, ASP pavement has a flat and stable metal layer with good conductivity of electric charge or heat (above the insulation layer), which will provide the opportunity for developing other novel pavement systems. For instance, a steel plate can be used as a thermally conductive layer for snow melting in winter, or a thermoelectric pavement for energy harvesting for instance [79], of solar heat. At the same time, prefabrication in the factory can directly install the sensors in the flat and stable steel plate or in a plastic layer of the pavement, which will improve the conversion efficiency and design life of the road for energy harvesting. Generally speaking, the construction cost of this new pavement structure will increase in the early stage, but its construction period is short, the construction variability is small and it has the characteristics of good performance, recyclability, environmental protection, etc., so as to achieve the goal of long-life pavement.

## 6. Conclusions and Further Study

I. Road pavement made using traditional civil engineering materials have the problem of material degradation in the long service period. At the same time, aggregates and asphalt are non-renewable resources; the mining and construction processes harm the environment. Due to the requirements for mechanical properties and durability, there are demands for new materials, to improve durability and construction efficiency and reduce environmental footprint.
II. ASP pavement structure was proposed in this paper, which uses the high-performance asphalt mixture (SMA, PA) as the surface functional layer, and the steel plate (A656) and plastic materials (ABS, POM and PET) as the load-bearing layer, replacing traditional bitumen-cound or cement-bound materials.
III. The ASP pavement (with a structure of 8 cm SMA asphalt mixture, 0.8 cm steel plate, 14 cm ABS and 20 cm crushed stones) has satisfactory mechanical properties and the surface deflection is similar to that on the traditional long-life pavement (with 35 cm asphalt layer overlaying 40 cm graded crushed stones).
IV. The GFRP insulation layer can retard the heat transfer downward from the surface effectively in ASP pavement. When the paving temperature of asphalt mixture reaches 170 °C, the peak temperature of the ABS upper surface or the GFRP insulation lower surface is about half of that paving temperature, which ensures that the plastic materials are in a safe and stable temperature range.
V. The steel plate and plastic materials in ASP pavement have good temperature stability and deformation resistance in the expected temperature range of pavement. The dynamic stability of the ASP slab specimens (with a size of 300 mm length × 300 mm width × 100 mm thickness, and a structure of 70 mm SMA, 5 mm steel plate, 5 mm GFRP and 20 mm ABS) can reach 10,000 times/mm.
VI. Due to the homogeneity and integrity of ABS and steel plate materials, and the high elastic modulus of steel plate, ASP pavement has a high elastic ratio and better fatigue resistance. Under the repeated loading of 1000 μm stress level, the fatigue life of the ASP beam specimen reaches about 250,000 times, which is twice as much as that of traditional SMA specimen.
VII. The main load-bearing layers in ASP pavement can be prefabricated and assembled on-site, which can improve the construction efficiency, quality and recyclability. ASP pavement has advantages over traditional pavement, including resource efficiency, durability and recyclability construction. Many of these benefits need to be quantified or verified by cost and environmental life cycle analysis.
VIII. This paper puts forward a new design concept for a novel pavement. However, as a new type of pavement structure, many more studies need to be carried out, before it can be accepted for practical use on things such as material specification, design criteria and method, mechanical characteristics of road structure, construction technology, long-term observation and evaluation, maintenance and rehabilitation methods.
IX. The biggest challenge of the ASP pavement is the fact that the cost increases compared with the traditional pavement. In future research, we should find alternative materials to reduce the cost.

## Figures and Tables

**Table 1:** The gradation of the graded gravel.

Mixture	Passing (by Mass) under Different Sieve Size (mm) (%)							
	31.5	19	16	9.5	4.75	2.36	0.6	0.75
Graded Gravel	100	-	70~90	50~70	40~60	25~40	20~32	8~15

**Table 2:** Properties of SBS modified asphalt binder.

Asphalt Binder	Penetration (25 °C) (0.1 mm)	Softening Point (°C)	Ductility (5 °C) (cm)	Dynamic Viscosity (60 °C) (Pa⋅s)
SBS	64	94.2	45.7	14,169.2

**Table 3:** Gradation of SMA for testing.

Mixture	Passing (by Mass) under Different Sieve Size (mm) (%)										Binder Content (%)
	16	13.2	9.5	4.75	2.36	1.18	0.6	0.3	0.15	0.075	
SMA-13	100.0	91.0	66.7	32.5	17.8	16.1	14.9	13.5	13.0	10.6	5.6

**Table 4:** The thickness of the ASP pavement structure.

Materials	Thickness, cm
SMA-13	4, 6, 8
Steel-plate	0.6, 0.8, 1.0, 1.2
ABS	12, 14, 16, 18, 20
Graded gravel	20
Subgrade	200

**Table 5:** The thickness of typical asphalt pavement structure.

Materials	Thickness, cm
SMA-13	5
Superpave mixture	25
ATB-30	5
Graded gravel	40
Subgrade	200

Note: ATB denotes asphalt treated base.

**Table 6:** Mechanical parameters of pavement materials are calculated by the finite element method [72,73,74].

Materials	Elastic Modulus (MPa)	Poisson’s Ratio	Density (g/cm^3^)
SMA-13	4500	0.35	2.40
Superpave Mixture	4500	0.35	2.40
ATB-30	3500	0.35	2.40
Steel plate	206,000	0.29	7.85
ABS	2200	0.39	1.05
Graded gravel	350	0.40	2.20
Subgrade	80	0.45	1.90

**Table 7:** Comparison of the mechanical properties of ASP and traditional pavement.

Index	ASP ^a^		Traditional Pavement
	Calculated Values	Allowable Values	
Tensile strain at the bottom of asphalt mixture layer ^b^ (με)	20.5	-	76.2
Tensile stress at the bottom of asphalt mixture layer ^b^ (MPa)	0.276	-	0.32
Tensile strain of steel plate (με)	7.1	844	-
Tensile stress of steel plate (MPa)	2.6	174	-
Tensile strain of ABS (με)	98.3	11,100	-
Tensile stress of ABS (MPa)	0.4	24.5	-
Vertical compressive strain on top of the subgrade (με)	138.5	-	83.9
Surface deflection (0.01 mm)	0.37	-	0.28

^a^ ASP pavement is 14-0.8-8, which includes an 8 cm asphalt mixture layer, 0.8 cm steel plate layer, 14 cm ABS and 20 cm graded crushed gravel layer. ^b^ The asphalt mixture layer of ASP pavement is 8 cm thick, and the structural correlation index is 35 cm thick asphalt mixture layer.

**Table 8:** Dynamic stability of ASP slab specimens.

Dynamic Stability (Times·mm^−1^)		Rutting Deformation (mm)	
Average Value	Standard Deviation	Average Value	Standard Deviation
10,414	122	2.58	0.04

**Table 9:** Comparison between ASP pavement and traditional pavement.

Contrastive Terms		ASP Pavement	Traditional Pavement
Efficiency	Automation in manufacturing	High	Low
	Prefabricated	Possible	Not possible
	Pavement thickness and weight	Thin/small	Thick/large
Durability	Material uniformity	Good	Poor
	Temperature effect	Low	High
	Crack resistance	Good	Medium
	Effect of humidity/water	Low	High
	Service life	Long life expectancy	20 or 40 years
Environmental protection	Recovery rate	High	Medium
	Impact on natural resources	Small	Large
	Construction emissions	Small	Large

## Data Availability

Access to any other materials can be requested by writing to the corresponding authors.

## Abbreviations

ASP	Asphalt steel plastic
SMA	Stone mastic asphalt
ABS	Acrylonitrile butadiene styrene
GFRP	Glass fiber reinforced polymer
SBS	Styrene-butadiene-styrene
FEA	Finite element analysis
POM	Polyoxymethylene
PET	Polyethylene terephthalate
HRA	Hot rolled asphalt
PA	Porous asphalt
ATB	Asphalt treated base
